# Optical Microsystem for Analysis of Diffuse Reflectance and Fluorescence Signals Applied to Early Gastrointestinal Cancer Detection

**DOI:** 10.3390/s150203138

**Published:** 2015-01-30

**Authors:** Sara Pimenta, Elisabete M. S. Castanheira, Graça Minas

**Affiliations:** 1 Department of Industrial Electronics, University of Minho, Campus de Azurém, Guimarães 4800-058, Portugal; E-Mail: gminas@dei.uminho.pt; 2 Centre of Physics (CFUM), University of Minho, Campus de Gualtar, 4710-057 Braga, Portugal; E-Mail: ecoutinho@fisica.uminho.pt

**Keywords:** optical microsystem, gastrointestinal cancer, early diagnostic, diffuse reflectance, fluorescence, mathematical models

## Abstract

The detection of cancer at its earliest stage is crucial in order to increase the probability of a successful treatment. Optical techniques, specifically diffuse reflectance and fluorescence, may considerably improve the ability to detect pre-cancerous lesions. These techniques have high sensitivity to some biomarkers present on the tissues, providing morphological and biochemical information of normal and diseased tissue. The development of a chip sized spectroscopy microsystem, based on these techniques, will greatly improve the early diagnosis of gastrointestinal cancers. The main innovation is the detection of the spectroscopic signals using only few, but representative, spectral bands allowing for miniaturization. This paper presents the mathematical models, its validation and analysis for retrieving data of the measured spectroscopic signals. These models were applied to a set of phantoms clearly representative of gastrointestinal tissues, leading to a more accurate diagnostic by a pathologist. Moreover, it was demonstrated that the models can use the reconstructed spectroscopic signals based only on its extraction on those specific spectral bands. As a result, the viability of the spectroscopy microsystem implementation was proved.

## 1. Introduction

The detection of cancer at the dysplasia stage (before macroscopically visible changes occur on the tissues) is one of the most important goals of biomedical research. Gastrointestinal (GI) cancers are usually preceded by pre-cancerous changes and its early detection will increase the chances of a successful treatment to the patient [1].

A GI dysplasia is difficult to detect by the conventional visual inspection during endoscopy or colonoscopy, due to the lack of macroscopically easily visible changes on the tissues in the early stage of cancer [1,2]. A large number of biopsies are performed in order to increase the detection probability of these invisible lesions. However, biopsies are procedures with sampling errors (since the sample condition may not be representative of the tissue malignant stage), high cost and are invasive to the patients. Finally, their results are not immediately available, resulting in a delay of patient's treatment [1,2].

**Figure 1.**
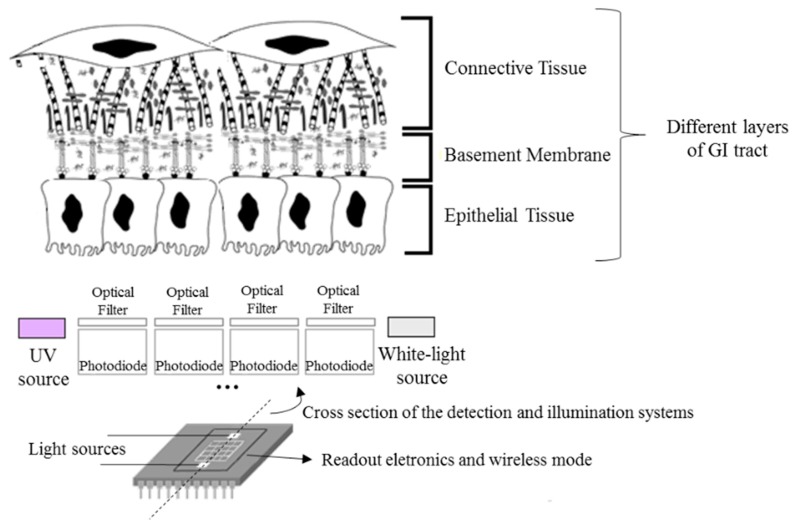
Miniaturized system to be implemented for illumination of gastrointestinal (GI) tissues and detection of diffuse reflectance and fluorescence signals in specific spectral bands, selected by the optical filters (not scaled, adapted [10,11]). Ultraviolet (UV) and white-light sources are required for fluorescence and diffuse reflectance measurements respectively.

As a result, there has been a growing interest in the study and development of new methods for the early and objective detection of GI cancer. Most of the GI cancers begin in the epithelium, the uppermost tissue layer (see Figure 1). Epithelial tissues acquire altered optical properties during cancer progression, especially due to the changes on their biochemical and morphological features. These small changes can be detected using spectroscopic techniques, especially diffuse reflectance and fluorescence. Spectroscopic techniques are based on light tissue interactions, allowing the detection of macroscopically invisible lesions on the tissue surface, lesions at the dysplastic stage [2–4].

Regarding diffuse reflectance, a white light is used for illumination of GI tissues and a spectrum of the reflected light is collected with information about tissue's optical properties [3]. The diffuse reflectance spectra are affected by absorption and scatter events. In the case of GI tissues, absorption is mainly due to the presence of hemoglobin (Hb), while scattering is caused by the collagen fibers present in the connective tissue [2–4]. Significant biochemical and morphologic changes are associated with cancer progression that may affect the intensity and shape of diffuse reflectance spectra. An increase of Hb concentration, related to angiogenesis, results in an increase of the tissue absorption coefficient and, consequently, in a reduction of the diffuse reflectance signal. Moreover, during cancer progression the epithelial thickness increases, which reduces the quantity of light that reaches the collagen fibers at connective tissue, decreasing the tissue scattering coefficient and, consequently, the diffuse reflectance signal intensity [2,3].

Concerning the fluorescence signal, a narrow spectral band of a light beam is used to excite fluorophores and the emission fluorescence spectra, at each excitation wavelength, are detected [3]. GI tissues produce fluorescence when excited by ultraviolet (UV) or blue wavelength visible light, since they have multiple fluorophores with excitation properties at this spectral band. The most significant markers of pre-cancerous changes are the collagen and the NADH (reduced form of nicotinamide adenine dinucleotide), which are related with cell structure and metabolism, respectively [2,3,5]. A decrease of collagen concentration and an increase of NADH concentration could be associated with cancer progression, resulting in a change in the intensity and shape of the fluorescence signal [3].

Therefore, the diffuse reflectance and fluorescence spectra can be used to extract information about tissue's malignancy degree, since they are dependent on the tissues biochemical and morphological state.

Several authors [1,5–7] have performed studies and have developed prototypes that include spectroscopy measurements for the detection of GI dysplasia. However, most of them use expensive, complex and bulky spectroscopy systems (xenon lamps, UV lasers, optical fibers and high quantum efficiency detectors), which may hamper its integration on endoscopic equipment. Moreover, most of the authors only consider the use of one spectroscopy signal (diffuse reflectance or fluorescence) or the use of a single molecule to study the fluorescence signal [8], which may not be enough for a correct and precise GI tissue characterization.

Other authors have tried to miniaturize their spectroscopy systems, by replacing the high quantum efficiency detectors by photodiodes [9]. However, they still use macroscopic equipment (monochromator and optical fibers) for illumination. Finally, Lo, *et al.* [4] developed a system with LEDs (Light Emitting Diodes) to extract and analyze the diffuse reflectance signal of phantoms. Despite this great advance towards miniaturization, the authors only proved their system viability to obtain information about tissues' absorption properties, which may not be enough to extract complete information for diagnosis of GI dysplasia.

As a result, the development of a spectroscopy microsystem on a chip that might be used *in-loco*, will have a high clinical value and represents the main innovative factor of the project under this paper. The microsystem will be portable and could be integrated in the conventional endoscopes or colonoscopes, for example, working as an auxiliary in GI tissues characterization and allowing the reduction of the early detection of GI dysplasia limitations. Moreover, the microsystem could be used in the surgery room for inspecting total removing of cancer tissue.

The microsystem (Figure 1) will combine two optical techniques—diffuse reflectance and fluorescence—and a matrix of thin-film optical filters deposited on silicon photodiodes, in order to select the relevant spectral bands to extract spectroscopic signals and detect GI dysplasia. Moreover, miniaturized LEDs will be incorporated on the chip, featuring illumination sources for diffuse reflectance and fluorescence measurements. Finally, readout electronics and a wireless mode also need to be considered, all integrated in a single chip, allowing data transmission and its analysis in a computer.

Before the microsystem implementation, it is crucial to study the behavior of the spectroscopy signals during cancer progression, specifically, the most important cancer biomarkers whose concentration changes during cancer progression and how its variation affects the intensity and shape of the spectroscopy signals. Moreover, it is also important to evaluate if the signals are not affected by other variables, such as the temperature of the tissue.

As a result, this paper describes the implementation of mathematical models, employed to retrieve the information of the measured spectroscopic signals, its validation and analysis using spectroscopic signals extracted only in a few spectral bands, proving the viability of using these signals to detect and interpret small changes on the tissues and, consequently, the future microsystem implementation. Spectroscopic measurements using phantoms representative of GI tissues were also performed in order to extract the experimental diffuse reflectance and fluorescence signals, used in the implemented models. Moreover, the effect of the tissues temperature in the spectroscopic signals intensity and shape was also evaluated.

## 2. Materials and Methods

**Figure 2.**
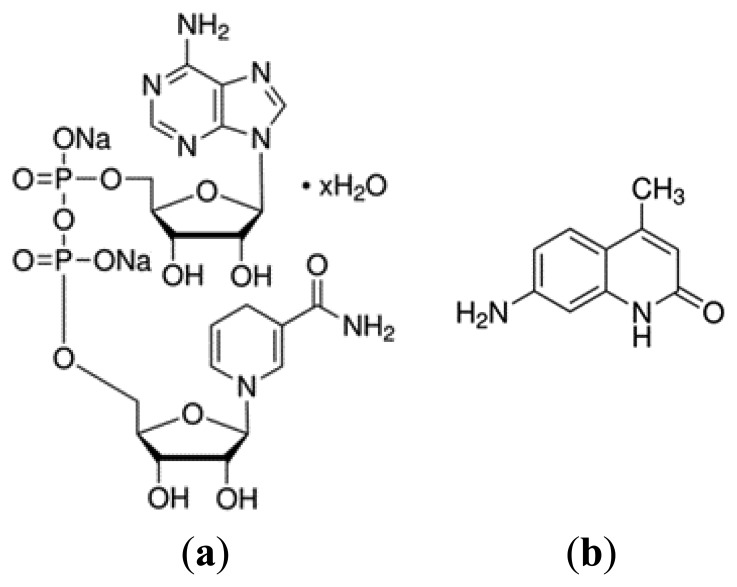
Structures of the fluorophores (**a**) NADH (reduced form of nicotinamide adenine dinucleotide) and (**b**) Carbostyril 124 (7-amino-4-methyl-2(1*H*)-quinolinone).

For the experimental tests presented in this paper, a set of liquid homogeneous phantoms representative of GI tissues was considered, with different concentrations of Hb, polystyrene beads (1 μm diameter), and the fluorophores NADH and Carbostyril 124 (7-amino-4-methyl-2(1*H*)-quinolinone, representative of collagen). The structures of both fluorophores are shown below (Figure 2). For each phantom created, its diffuse reflectance signal was obtained, between 350 nm and 750 nm, with a commercial UV-Vis-NIR spectrophotometer (Shimadzu UV 3101PC) equipped with an integrating sphere. The fluorescence signal was obtained, between 380 nm and 600 nm (with excitation at 350 nm), using a commercial fluorometer (SPEX^®^ FluoroLog^®^ 2), equipped with a temperature controlled cuvette holder. Temperature was kept constant at the chosen value ±0.2 °C. All the fluorescence spectra were corrected for the instrumental response of the equipment (using the correction curve provided by the manufacturer). The influence of intensity fluctuations of the lamp was eliminated by the acquisition system (dividing the sample signal by a reference signal acquired by a photodiode). Therefore, fluorescence signal has no variability for each of the samples at a given temperature.

## 3. Results and Discussion—Characterization and Analysis of Spectroscopic Signals

**Figure 3.**
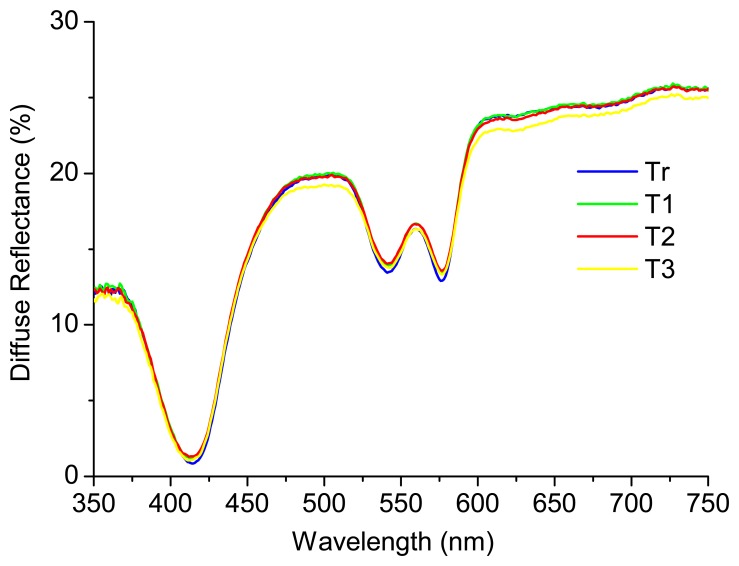
Diffuse reflectance spectra at different temperatures for a test phantom.

A recent study performed by the research team [12], where phantoms representative of only absorption and scattering properties were considered, concluded that the diffuse reflectance signal is not affected by the temperature of the sample. Figure 3 shows an example of a phantom (liquid homogeneous phantom with Hb concentration of 1 mg/mL (absorber) and intralipid mass concentration of 0.5% (scatterer)) at four different temperatures: Tr (room temperature (22 °C)), T1 (37 °C), T2 (40 °C) and T3 (42 °C). As it can be seen, the diffuse reflectance signal is similar for all the temperatures tested. The results obtained were analyzed in SPSS software, through a partial correlation, for accurately checking the relation between temperature and diffuse reflectance signal intensity, controlling for wavelength and absorber and scatterer concentrations. The partial correlation coefficients were used in this statistical analysis and a *p*-value < 0.05 was considered statistically significant. The results obtained allow concluding that the temperature does not have a statistically significant affectation (*r* (3204) = −0.004, *p* > 0.05) in the diffuse reflectance spectra, in the range of temperatures tested (22 °C to 42 °C).

In the same study [12], the authors implemented a Monte Carlo based inverse model in order to extract tissues optical properties (absorption and reduced scattering coefficients—μ_a_ and μ_s_', respectively) based on the measurement of the diffuse reflectance signal. μ_a_ is related with the concentration of chromophores in the tissue, such as hemoglobin. μ_s_' is related with the size and concentration of scattering molecules, such as collagen fibers in the epithelial tissues. Small changes in these coefficients may be associated with cancer progression, as explained in Section 1. In that study [12], a set of liquid homogeneous phantoms was considered, with variable concentrations of an absorber (Hb) and a fixed concentration of a scatterer (intralipid). Thus, the phantoms were only representative of absorption and scattering properties.

In this paper, phantoms representative of absorption, scattering and also fluorescence properties were used, considering all markers of GI pre-cancerous changes. As a result, a set of liquid homogeneous phantoms with different concentrations of Hb, polystyrene beads, and the fluorophores NADH and Carbostyril 124 was created (Table 1). In this phase, besides using a more representative group of phantoms, it was also used a different scatterer, the polystyrene beads (to represent the collagen fibers of GI tissues), since intralipid exhibits fluorescence emission when excited with ultraviolet (UV) or blue wavelength visible light, which may hamper the interpretation of information extracted from the fluorescence signal.

The model previously implemented and described in detail in [12] was applied to these phantoms in order to extract μ_a_ and μ_s_' values that characterize them, based on their experimental diffuse reflectance signal. The model is based on a established equation [13] that relates the diffuse reflectance of a tissue at each wavelength—R_m_(*λ*), with its optical properties as a function of wavelength—μ_a_ (*λ*) and μ_s_'(*λ*)—Equation (1).

(1) $$R_{m}\left(\lambda \right)=\sum_{j=1}^{N}N_{\text{reflect}}\left(j\right)\left(\frac{c\left(\lambda \right)}{c_{\text{sim}}\left(\lambda \right)}\right)j$$

where *N* represents the mean number of interactions between each photon (that exits the tissue surface) and the medium; *N*_reflect_ (*j*) is the portion of reflected photons after j interactions with the medium; *c*(*λ*) is a ratio (obtained with μ_a_ and μ_s_', as detailed in [12]) that defines the optical properties of the tissue; and *c*_sim_(*λ*) is a ratio that defines the optical properties of a reference phantom. The diffuse reflectance of the reference phantom (obtained with the Monte Carlo forward model [14]) will be used for normalizing the experimental diffuse reflectance of the tissue. Moreover, a Matlab optimization function, *lsqcurvefit*, is also used with initial random input solutions for absorption and reduced scattering coefficients (*c*(*λ*)). This function is based on the least-squares algorithm and its main goal is to iteratively update *c*(*λ*), until the value of modulated reflectance (Equation (1) output) is similar to the experimental diffuse reflectance that defines our phantom. In other words, the function finds the best absorption and reduced scattering coefficients (μ_a_ and μ_s_') that fit a specific diffuse reflectance spectrum. All the theoretical considerations used in the implemented model are explained in [12].

**Figure 4.**
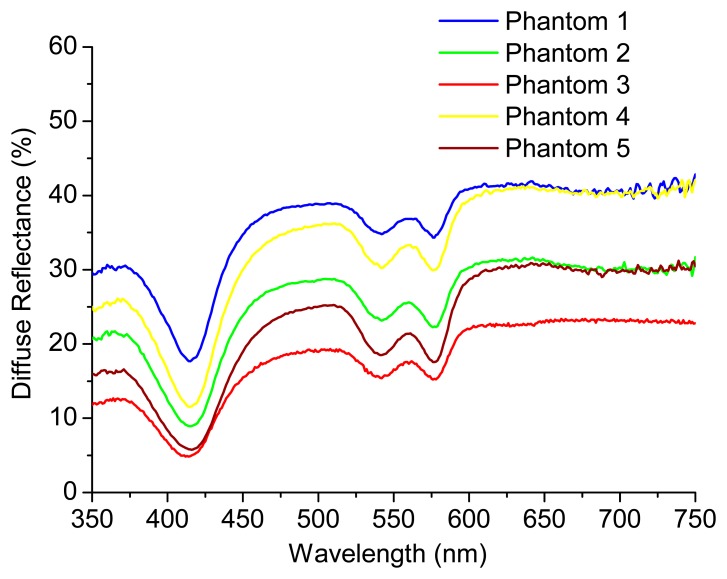
Experimental diffuse reflectance signal for all the test phantoms.

**Figure 5.**
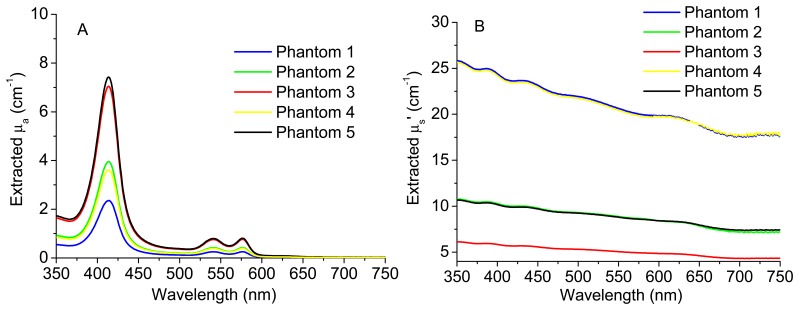
Extracted absorption (**A**) and reduced scattering (**B**) coefficients for all the test phantoms.

The knowledge about μ_a_ (*λ*) and μ_s_' (*λ*) can be used by the pathologist to detect small changes in these coefficients that could be related with cancer progression. Figure 4 shows the experimental diffuse reflectance spectra for all the phantoms presented in Table 1. Figure 5A,B exhibits the extracted absorption and reduced scattering coefficients for these phantoms, respectively, as a function of wavelength.

As can be observed in Figure 5A, the extracted absorption coefficient, μ_a_, of phantom 1 is lower than those of all the other phantoms, since phantom 1 has the lowest Hb concentration. Moreover, phantoms 3 and 5 present a similar μ_a_ value as they have the same Hb concentration. The same happens for phantoms 2 and 4. Concerning Figure 5B, phantom 3 has the lowest polystyrene mass concentration and, for this reason, the lowest value of the reduced scattering coefficient, μ_s_'. Finally, since the polystyrene mass concentration is the same for phantoms 2 and 5, they have similar μ_s_' values. The same happens for phantoms 1 and 4.

**Figure 6.**
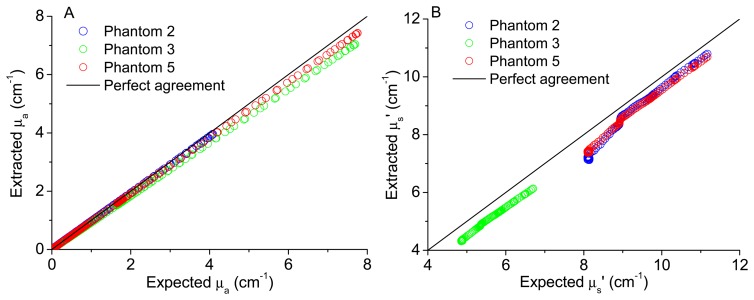
(**A**) Extracted versus expected absorption coefficient (μ_a_ (cm^−1^)) for three of the test phantoms; (**B**) Extracted versus expected reduced scattering coefficient (μ_s_' (cm^−1^)) for three of the test phantoms.

In order to validate the implemented model to extract tissues optical properties, the comparison between the extracted and expected coefficients was performed. Figure 6A,B shows the plots of the extracted *versus* expected absorption and reduced scattering coefficients, respectively, for all wavelengths and for three of the test phantoms (phantoms 2, 3 and 5). These phantoms were chosen because phantoms 3 and 5 have the same absorption coefficient but different scattering coefficients, while phantoms 2 and 5 have the same scattering coefficient but different absorption properties. Choosing these features, the model capacity of reproducibility can be observed for different phantoms sharing the same absorbent or scattering properties. The expected (theoretical) coefficients were obtained considering the known features of the phantoms created and by application of Equation (2), to obtain the expected μ_a_ (*λ*), and Mie theory for spherical particles, to obtain the expected μ_s_' (*λ*), available as free software in [15], as explained in detail in [12],

(2) $$\mu_{a}\left(\lambda \right)=\ln\left(10\right)\times \varepsilon_{i}\left(\lambda \right)\times c_{i}$$

where ε_i_(*λ*) is the extinction coefficient of the absorber (Hb), that defines its capacity to absorb light as a function of wavelength; and *C*_i_ is the Hb concentration in the phantom.

**Figure 7.**
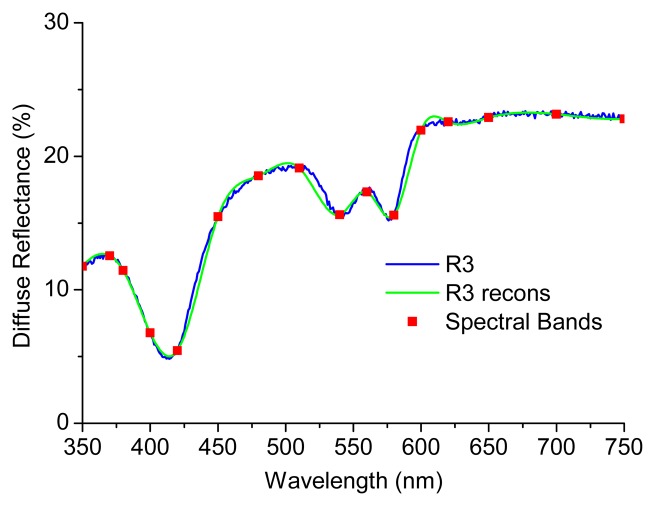
Diffuse Reflectance from phantom 3: Experimental spectra (R3), reconstructed spectra (R3 recons) and the 16 spectral bands used for reconstruction (Spectral bands).

It is important to note that for the coefficients extraction it were used the reconstructed spectra of each phantom by the application of a *spline* Matlab function and based only in 16 values of diffuse reflectance (corresponding to the same spectral bands previously considered appropriate to GI malignancy detection, as referred in [10,16]). In fact, this is the main innovation of the microsystem presented in Figure 1, the extraction of only a few values of diffuse reflectance signal to extract tissues properties and, consequently, to detect small changes. Figure 7 shows the experimental diffuse reflectance spectra (blue curve—R3) and the reconstructed diffuse reflectance spectra (green curve—R3 recons) for phantom 3, based on the use of 16 spectral bands (red points—spectral bands) and a Matlab *spline* function.

As it can be observed in Figures 5 and 6, the use of the reconstructed diffuse reflectance spectra (from the 16 spectral bands) in the implemented inverse model allows the extraction of the absorption and scattering coefficients with low differences between the expected and extracted tissue optical properties. Thus, the use of the reconstructed diffuse reflectance signal to detect changes indicative of cancer progression on the tissues is validated.

**Figure 8.**
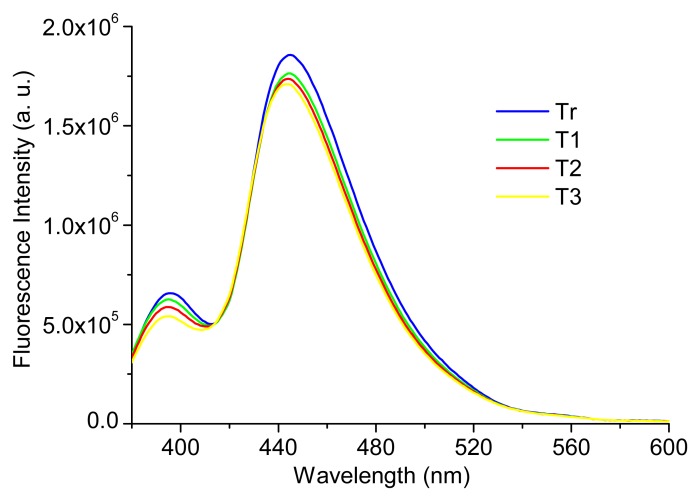
Fluorescence spectra (λ_exc_ = 350 nm) at different temperatures for phantom 2, described in Table 1.

Concerning the fluorescence signal, experimental measurements were performed (*n* = 20) in order to evaluate the temperature dependence of the fluorescence signal, with the phantoms presented in Table 1, at four different temperatures: Tr (room temperature (22 °C)), T1 (37 °C), T2 (40 °C) and T3 (42 °C). Figure 8 shows the obtained results for phantom 2. As it was expected, there are slight differences between the fluorescence signals at different temperatures. In fact, an increase in temperature generally results in a decrease in the fluorescence intensity because the non-radiative deactivation processes, related with thermal agitation, are more efficient at higher temperatures [17]. Similar results were obtained for all the phantoms created (see Table 1). In spite of this, all the results obtained were analyzed in SPSS software, through a partial correlation, for checking the relation between temperature and fluorescence signal intensity, controlling both for wavelength and fluorophores concentration. The partial correlation coefficients were used in this statistical analysis and a *p*-value < 0.05 was considered statistically significant. The results obtained allow concluding that despite the slight differences, they are not statistically significant (*r* (3008) = −0.007, *p* > 0.05), in the range of temperatures tested (22 °C to 42 °C).

These results allow concluding that in the analysis of the fluorescence signal to extract information about tissues condition, it is not necessary to consider its temperature, since this factor will not have an effect statistically significant in the fluorescence intensity.

The fluorescence signal of a tissue (usually referred as bulk fluorescence) is affected by absorption and scattering events, which could introduce distortions in spectral intensity and shape, not allowing a correct identification of tissue fluorophores and its concentration changes on the tissue, which is usually related with cancer progression [2,18]. NADH has a maximum fluorescence emission between 450 nm and 460 nm, with a quantum efficiency of 2% [19], while Carbostyril 124 in solution presents a maximum emission wavelength close to 417 nm, with a quantum efficiency of 97% [20].

**Figure 9.**
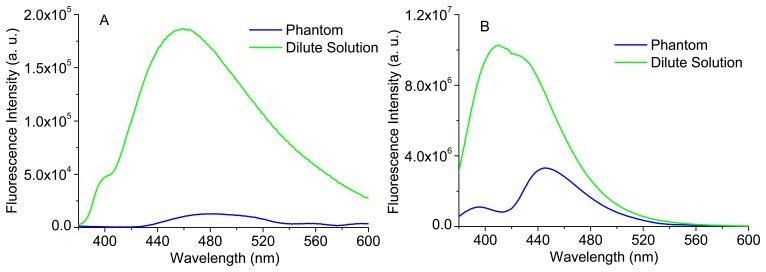
Comparison between the fluorescence emission spectra (λ_exc_ = 350 nm) of NADH (reduced form of nicotinamide adenine dinucleotide) 1.5 μg/mL (**A**) and Carbostyril 124 (7-amino-4-methyl-2(1*H*)-quinolinone) 1.5 μg/mL; (**B**) in homogeneous solution and in a phantom containing 1 mg/mL of Hb (hemoglobin) and 0.15% w/v (weight/volume) of polystyrene beads.

Figure 9 exhibits the comparison between the spectra of each fluorophore in diluted solution and in a phantom, containing Hb and polystyrene beads. It can be observed that, despite the same concentration of fluorophore, the spectra in turbid media (phantoms) are very different from the ones in homogeneous dilute solution, as the fluorescence emitted from the surface of a turbid medium may change from isotropic to anisotropic [21]. Besides the differences in intensity, due to the absorption and scattering effects in phantoms, the spectral shape is also affected, especially in the case of Carbostyril 124. The spectra in phantom (B) is very similar to the one reported for an arterial tissue sample [21], evidencing that the tissues fluorescence is dominated by collagen, due to the low quantum efficiency of NADH. The valley near 420 nm corresponds to an absorption band of Hb (the Soret band [21]).

**Figure 10.**
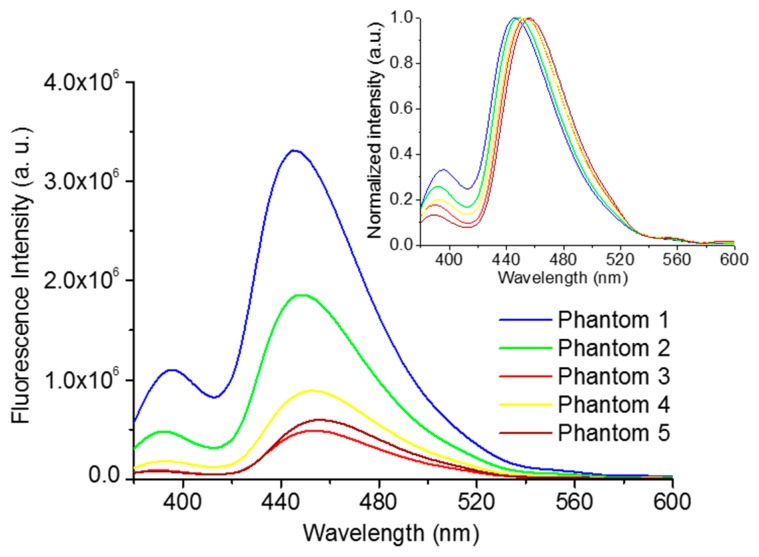
Bulk fluorescence spectra (λ_exc_ = 350 nm) for all phantoms created (Inset: Normalized spectra).

Figure 10 shows the bulk fluorescence spectra of each phantom presented in Table 1, being similar in shape to the phantom spectrum of Figure 9B, as expected. However, the ratio between the two bands (near 450 nm and 390 nm) is higher, due to the presence of NADH. For example, considering phantoms 1, 2 and 3, the ratio between the two bands is 3.02, 4.08 and 5.64, respectively, due to an increase in the NADH concentration along the phantoms 1 to 3 (see inset of Figure 10, the normalized fluorescence spectra).

However, it is important to note that the increase in the ratio between the two bands is not proportional to NADH concentration, since the fluorescence intensity and, consequently, the ratio values are affected by absorption and scattering events. Comparing the fluorescence intensity of phantoms 2, 4 and 5, for example, in spite of having the same fluorophores concentration, they have distinct spectra, due to the presence of different Hb and polystyrene beads concentrations. As expected, the fluorescence intensity decreases as the Hb concentration increases (Phantoms 2 and 5), due to an increase in absorption. The same happens when the concentration of polystyrene beads is increased, due to the enhancement of scattering (Phantoms 2 and 4).

As a result, the implementation of a model to extract the intrinsic fluorescence (fluorescence without diffusion or absorption distortions, only due to tissue fluorophores) from the bulk fluorescence (measured in a turbid media such as a phantom or tissue) is critical in order to detect with a higher accuracy the small changes on the tissues fluorophores concentration that may occur during cancer progression.

The implemented algorithm (in Matlab) is based on the photon migration model developed by Wu *et al.* [22] and modified by Zhang *et al.* [18] and Müller *et al.* [23]. This model allows extracting the intrinsic fluorescence (*f*) based on the bulk fluorescence (*F*) and diffuse reflectance (*R*) spectra of a tissue, by the application of Equation (3):

(3) $$f_{x,m}=\frac{F_{x,m}}{\frac{1}{\mu_{s,x}l}{\left(\frac{R_{0,x}R_{0,m}}{\left[e^{S\left(1-g_{x}\right)}-1\right]\left[e^{S\left(1-gm\right)}-1\right]}\right)}^{\frac{1}{2}}\frac{R_{x}}{R_{0,x}}\left(\frac{R_{m}}{R_{0,x}}+\left[e^{S\left(1-g_{m}\right)}-1\right]\right)}$$

where *x* and *m* denote the excitation and emission wavelengths; *R_x_* and *R_m_* correspond to the experimental diffuse reflectance at the excitation and emission wavelengths, respectively; *R*_0_,*_x_* and *R*_0_,*_m_* represent the diffuse reflectance that would be measured in the absence of absorption. These values were obtained by the application of a Monte Carlo forward model (freely available software [14]), considering the extracted scattering coefficient (μ_s_) for each phantom (Figure 5) and setting the absorption coefficient (μ_a_) to zero; μ_s,x_ is the extracted scattering coefficient at the excitation wavelength; *g_x_* and *g_m_* correspond to the anisotropic coefficient at excitation and emission wavelengths, respectively; *l* is the sample thickness; and (*S*) is a probe-specific constant.

**Figure 11.**
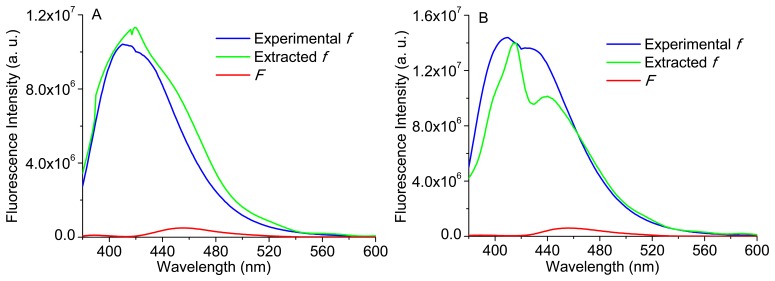
(**A**) Experimental and extracted intrinsic fluorescence (*f*) and bulk fluorescence (*F*) for phantom 3; (**B**) Experimental and extracted intrinsic fluorescence (*f*) and bulk fluorescence (*F*) for phantom 5.

Therefore, Equation (3) was implemented to some of the phantoms presented in Table 1. Again, it is important to note that the reconstructed bulk fluorescence spectra of each phantom were used, by the application of a *spline* Matlab function and based only in 10 discrete values of the bulk fluorescence signal at the wavelengths: 380, 400, 420, 450, 480, 510, 540, 560, 580 and 600 nm. Figure 11A,B shows the extracted intrinsic fluorescence for phantoms 3 and 5 (see Table 1), their experimental intrinsic fluorescence (*f*) and the bulk fluorescence (*F*). The experimental intrinsic fluorescence for each phantom was obtained using a sample with only Carbostyril 124 and NADH (in the same concentration of the respective phantom).

As it can be observed in Figure 11, the implementation of Equation (3) allows the extraction of the intrinsic fluorescence, based on the experimental reconstructed spectra (diffuse reflectance and bulk fluorescence of a phantom) and in the use of extracted scattering coefficients (μ_s_), by the implementation of a Monte Carlo based inverse model. Moreover, the modeled intrinsic fluorescence is similar to the experimental intrinsic fluorescence. Slight differences could be related with the use of the extracted coefficients to obtain *R*_0_,*_x_* and *R*_0_,*_m_* or with the use of the reconstructed diffuse reflectance and bulk fluorescence spectra (based only in a few spectral bands).

Analyzing the obtained spectra, the extracted intrinsic fluorescence spectrum of phantom 3 (Figure 11A) presents only one emission peak (near to 420 nm), similar to that of collagen in very thin tissues [18], since the fluorescence is dominated by Carbostyril 124 (representative of collagen), which has high quantum efficiency. However, the presence of a higher NADH concentration (comparing with phantom 5) is also responsible for the occurrence of a single and larger peak, because this is the characteristic effect of NADH in the emission fluorescence spectra [20]. Concerning the extracted spectrum of phantom 5 (Figure 11B), it has two emission peaks, and the right-side shoulder is more pronounced, which can indicate some aggregation of the Carbostyril 124 fluorophore. As the Carbostyril 124 concentration is higher, the spectral intensity is also higher (especially the first peak, which corresponds to the emission fluorescence peak of Carbostyril 124), but not proportional to its concentration.

**Figure 12.**
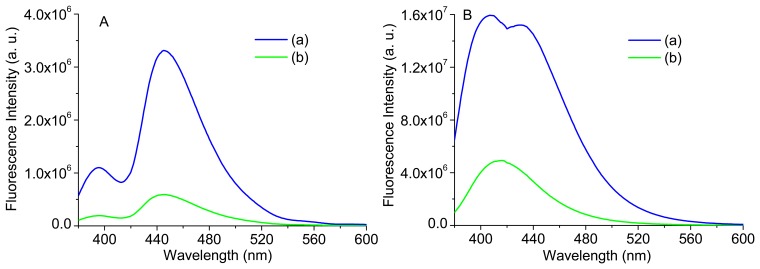
Bulk Fluorescence (**A**) and Intrinsic Fluorescence; (**B**) for two phantoms with different Carbostyril 124 concentrations: (a) 1.5 μg/mL; and (b) 0.15 μg/mL.

Figure 12 displays the bulk and intrinsic fluorescence of two identical phantoms; one with the same concentration of phantom 1 and the other with a concentration of Carbostyril ten times lower. It can be observed that, despite the small differences in shape of bulk fluorescence spectra, the intrinsic fluorescence is quite different, highlighting again the importance of extracting the intrinsic fluorescence in order to more accurately interpret the small changes on the tissues. The intrinsic fluorescence of the more diluted sample—B(b)—is in accordance to the reported fluorescence spectrum of Carbostyril 124 in dilute solution, with a maximum at 417 nm [20] and with the intrinsic fluorescence spectrum of phantom 3 (see Figure 11A), while the one with higher concentration—B(a)—is very similar to that of an optically thin arterial tissue [21] and with the intrinsic fluorescence of phantom 5 (see Figure 11B). Thus, it can be concluded that the spectral difference presented between the intrinsic fluorescence signals of phantoms 3 and 5 (Figure 11A,B, respectively), in intensity and shape, are in accordance with the differences in NADH and Carbostyril 124 concentrations.

In this section, it was proved the possibility to retrieve data from the measured spectroscopic signals (bulk fluorescence and diffuse reflectance signals) using only a few spectral bands (in this case, 16), validating the future microsystem implementation. The validation was performed with a set of phantoms with known features, in order to perform a complete and precise validation of the implemented models, since it was possible to compare the experimental results with the ones that were expected, based on the known features of the phantoms created.

In a future step of the work (after the microsystem implementation) *ex vivo* tests will be performed, extracting the intrinsic fluorescence signal of unknown human GI tissues (the microsystem will extract the bulk fluorescence that will be used to obtain the intrinsic fluorescence) and comparing with the typical intrinsic fluorescence signal of a normal human GI tissue (at that stage, the help of a pathologist will be essential). Moreover, statistical tests will also be carried out in order to achieve a quantitative comparison between the signals, allowing concluding if there are significant differences between the normal tissue and the sample tissue. If that occurs, once the intrinsic fluorescence signal has only information about fluorophores (by the application of the model presented in this section), it will be possible to extract quantitative information about the biochemical tissue composition performing a simple linear decomposition. This information, together with the quantitative data extracted from the diffuse reflectance signal (absorption and scattering coefficients), which will be also measured by the microsystem, will help the pathologist to obtain a more accurate and complete tissue diagnosis.

## 4. Conclusions

In this paper, a mathematical model previously implemented to extract tissues optical properties (absorption and reduced scattering coefficients), based on the diffuse reflectance signal, was validated with a set of phantoms, which are clearly representative of GI tissues. As a follow-up of the work, another mathematical model was implemented to extract the intrinsic fluorescence based on bulk fluorescence, diffuse reflectance and extracted coefficients from the first implemented model. As a result, it was possible to extract phantoms spectroscopic signals and use them to obtain the tissues properties related not only with absorption and scattering, but also with fluorophores concentration. This knowledge can be used by a pathologist to accurately detect small changes in these coefficients, which could be related with biochemical and morphological changes on the tissues during cancer progression. Since the models use the reconstructed spectroscopic signals based only on their extraction on specific spectral bands, the future microsystem implementation is validated.

Finally, spectroscopic measurements in phantoms at different temperatures allow to conclude that in the analysis of the diffuse reflectance and fluorescence signals it is not necessary to consider the sample temperature, since this factor will not have a statistically significant effect in signals shape and intensity, validating the future microsystem application *ex vivo* or *in vivo*.

## Figures and Tables

**Table 1.** Phantoms used for the validation and analysis of spectroscopic signals.

**Phantom**	**Hb Concentration (mg/mL)**	**Polystyrene Mass Concentration (%)**	**NADH Concentration (μg/mL)**	**Carbostyril Concentration (μg/mL)**

**1**	0.25	0.50	0.50	1.50
**2**	0.50	0.25	1.00	1.00
**3**	1.00	0.15	1.50	0.50
**4**	0.50	0.50	1.00	1.00
**5**	1.00	0.25	1.00	1.00
